# Vascular Reconstruction of Multiple Renal Arteries—A Risk Factor for Transplant Renal Artery Stenosis: Insight From a Matched Case-Control Study

**DOI:** 10.3389/ti.2024.13298

**Published:** 2024-11-07

**Authors:** Devprakash Choudhary, Rajesh Vijayvergiya, Kamal Kishore, Vanji Nathan Subramani, Mohan Banoth, Sai Praneeth Reddy Perugu, Milind Mandwar, Bharat Bamaniya, Arun Panjathia, Parul Gupta, Shiva Kumar S. Patil, Jasmine Sethi, Ujjwal Gorsi, Sarbpreet Singh, Deepesh Kenwar, Ashish Sharma

**Affiliations:** ^1^ Department of Renal Transplant Surgery, Post Graduate Institute of Medical Education and Research (PGIMER), Chandigarh, India; ^2^ Department of Cardiology, PGIMER, Chandigarh, India; ^3^ Department of Biostatistics, PGIMER, Chandigarh, India; ^4^ Department of Hospital Administration, Post Graduate Institute of Medical Education and Research (PGIMER), Chandigarh, India; ^5^ Department of Nephrology, PGIMER, Chandigarh, India; ^6^ Department of Radiology, PGIMER, Chandigarh, India

**Keywords:** transplant renal artery stenosis, vascular reconstruction, multiple renal arteries, ex-vivo back-table reconstruction, endovascular intervention

## Abstract

Transplant Renal Artery Stenosis (TRAS) is the leading vascular complication following kidney transplantation (KT), causing premature allograft loss and increased post-KT mortality. While risk factors for TRAS, such as prolonged cold ischemia time and delayed graft function, are well-documented in deceased donor-KT, the risk factors remain less clearly defined in living donor-KT. This matched case-control study, conducted at a leading national transplant center predominantly performing living donor-KT, evaluated risk factors and long-term outcomes of clinical TRAS (cTRAS). cTRAS cases diagnosed from January 2009 to December 2022 were matched with four control kidney transplant recipients (KTRs) in a study powered to assess whether *ex-vivo* arterial vascular reconstruction of multiple renal arteries (VR-MRA) increases the risk of cTRAS. Among 2,454 KTs, 28 KTRs (1.14%) were diagnosed with cTRAS around 3.62 ± 1.04 months post-KT, with renal allograft dysfunction (92.86%) as the most common presenting feature. Notably, 27 cTRAS cases were successfully treated with endovascular intervention, yielding favorable outcomes over a 6–180 months follow-up period. The study identified *ex-vivo* VR-MRA as an independent risk factor for cTRAS (P < 0.001). cTRAS cases receiving timely treatment exhibited long-term outcomes in graft and patient survival similar to control KTRs. Early screening and timely intervention for cTRAS post-KT may improve graft and patient outcomes.

## Graphical Abstract

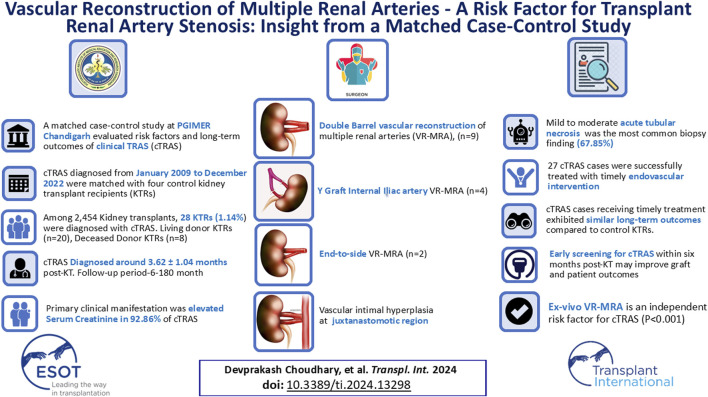

## Introduction

Kidney transplantation (KT) is the optimal therapy for individuals with end-stage renal disease (ESRD). With advancements in immunosuppression, non-immunological elements have emerged as the primary cause of allograft loss and mortality among kidney transplant recipients (KTRs). During the initial 6 months post-KT, surgical complications present a higher risk of allograft loss compared to allograft rejection [1]. Transplant Renal Artery Stenosis (TRAS) is the predominant vascular complication following KT, accounting for 75% of such issues. TRAS significantly contributes to allograft dysfunction, allograft loss, and premature death amongst KTRs [2]. The reversible nature of TRAS emphasizes the importance of prompt diagnosis and timely intervention to prevent irreversible allograft damage caused by TRAS, thereby reducing allograft loss and improving patient survival [3].

Since its first identification in 1973, varying diagnostic criteria and improved screening techniques have resulted in a reported increase in the incidence of TRAS from 1% to 23% post-KT [3–5]. However the majority of risk factors associated with TRAS were described concerning deceased donor-KT, like expanded criteria donors (ECD), older donors and recipients, prolonged cold ischemia time (CIT), delayed graft function (DGF), allograft-rejection, diabetes-mellitus (DM), and atherosclerotic vessels [3]. Nevertheless, these risk factors are much less prevalent in living donor-KT.

In a series of clinical-TRAS (cTRAS) following living donor-KT, the utilization of internal iliac artery Y-graft for allografts with multiple renal arteries (MRA) has been suggested as a potential risk factor for cTRAS, indicating a complex interaction of anatomical variations and vascular reconstruction surgical techniques affecting the risk of cTRAS in living donor-KT [6]. This matched case-control study, conducted at a leading national transplant center known for primarily performing living donor-KT, was designed to identify various risk factors and outcomes associated with cTRAS. The present study hypothesized that *ex-vivo* back-table vascular reconstruction of multiple renal arteries (VR-MRA) could significantly contribute to the development of cTRAS by inducing vascular intimal hyperplasia (IH) at the juxtanastomotic region. This IH could disproportionately affect the luminal diameter, particularly in the reconstructed smaller vessels of multiple renal arteries, thereby providing a biological rationale for the occurrence of cTRAS.

## Patients and Methods

### Study Population

This study involving data from human participants was approved by the Institutional Ethical Committee of the Post Graduate Institute of Medical Education and Research (PGIMER), Chandigarh, INDIA (NK/7617/study/710). The research adhered to the ethical standards outlined in the 1964 Declaration of Helsinki and its subsequent amendments or comparable ethical standards. Considering the retrospective nature of the study, the informed consent was waived by the PGIMER ethics committee. The study included KT performed from January 2009 to December 2022. The follow-up duration extended from the date of KT to December 2023.

### Cases and Controls

Cases and controls were selected from the study center’s prospectively maintained electronic database. The cases comprised KTRs diagnosed with cTRAS. The matched control encompassed KTRs who underwent KT within the same calendar year but did not develop cTRAS.

### Matching Criteria and Control Allocation

Each TRAS case was matched with four control KTRs using nearest neighbor matching to control for confounding factors. Subject in the cTRAS group was matched with the nearest control subject (KTRs) transplanted by the same surgeon in the same year the cTRAS case was diagnosed based on observed characteristics. The matching criteria included time from KT, senior operating surgeon, KTR age (within ±5 years), KTR gender, KT timing, and type of transplant (living vs. deceased donor). By maintaining consistency in the operating surgeon and these various parameters, we aimed to minimize bias and ensure comparability between the groups.

### cTRAS Definition

cTRAS observed post-KT was identified following an elevation in serum creatinine (Scr-mg/dL) by over 20% from baseline or the presence of symptoms, including reduced urine output, fluid retention, weight gain, or worsening uncontrolled hypertension requiring more than one antihypertensive medication after ruling out other causes of allograft dysfunction, such as allograft-rejection, infection, drug-toxicity, acute kidney injury, or recurrence of primary disease. The diagnosis of cTRAS is then confirmed through selective renal angiography following supportive color Doppler ultrasound (CDU) findings.

*Positive CDU criteria* included a renal artery peak systolic velocity of ≥200 cm/s and/or distal spectral broadening or a parvus tardus waveform with a low resistive index (<0.5) in post-stenotic intrarenal arteries.

Confirmatory angiographic evidence of cTRAS included renal artery stenosis >50% of the renal artery (RA) internal diameter with successful stenosis correction leading to an improved renal-allograft function and/or blood pressure regulation.

*Hypertension (HTN) post-KT* in KTRs was defined as blood pressure readings exceeding 130/80 mm Hg on more than two separate occasions. First-line agents included calcium channel blockers; second-line were thiazide diuretics and/or Beta-blockers or (ACE inhibitors or ARBs).

### Exclusion Criteria

KTRs who experienced immediate postoperative technical complications, such as RA dissection or kinking, requiring intervention within 1 month post-KT for renal artery stenosis, were excluded.

### Study Aim

To investigate the risk factors and outcomes associated with cTRAS amongst KTRs.

### Study Objective

To evaluate the association between VR-MRA and the heightened risk of cTRAS.

### Study Hypothesis

Performing VR-MRA to create a common channel for vascular implantation is associated with an increased risk of cTRAS.

### Study Parameters: Baseline Characteristics

Type of transplant (living donor-KT or deceased donor-KT), baseline donor and recipient demographics, pre-transplant hemodialysis duration, and HLA mismatch.

### Intraoperative Variables

**FIGURE 1:**
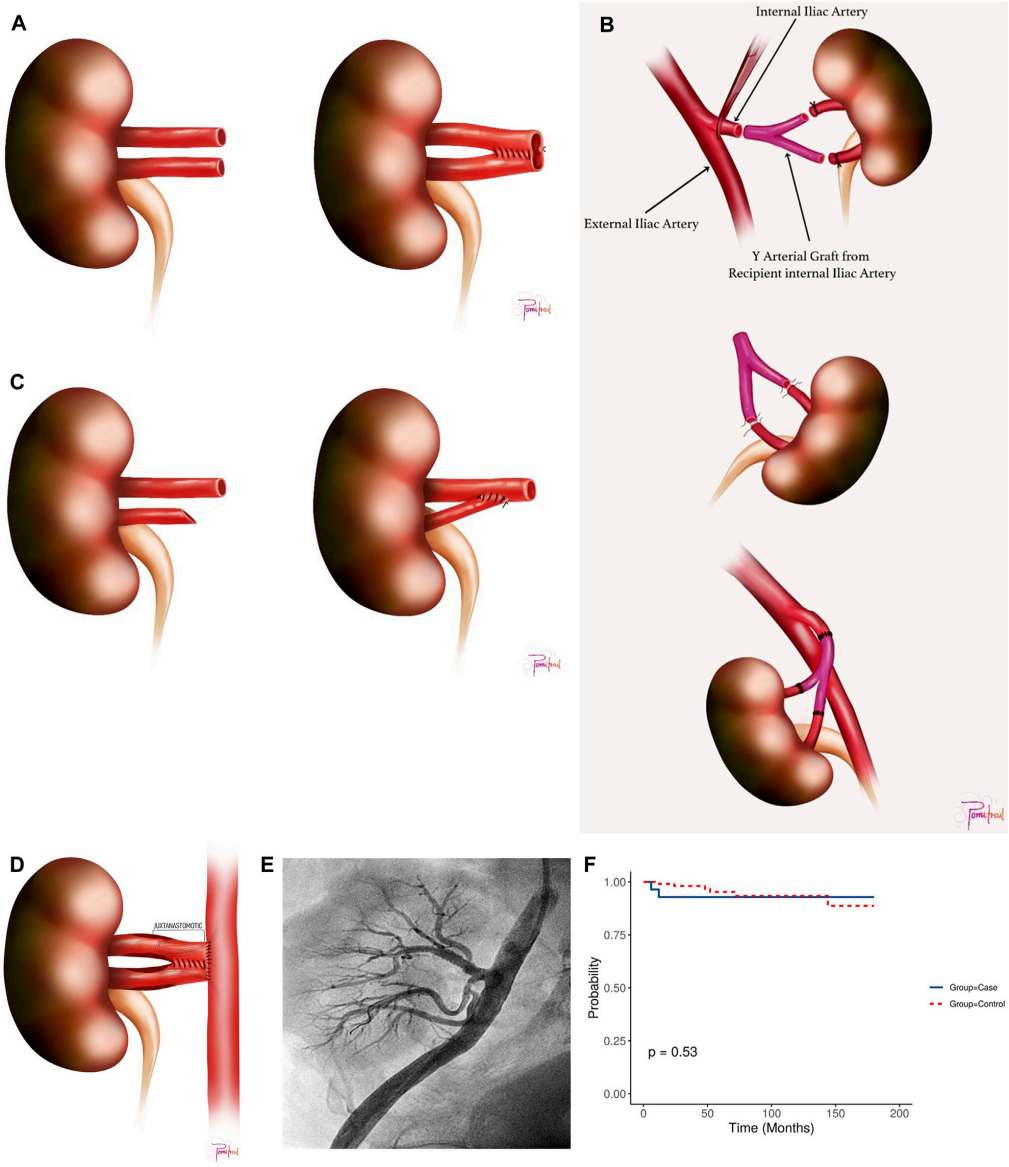
**(A)** Double Barrel/Pantaloon Technique. **(B)** Y Graft Internal Iliac Artery Technique. **(C)** End-to-side anastomosis of the smaller artery to the main artery. **(D)** Juxta-anastomosis Region with Pantaloon Technique. **(E)** Post-intervention angiography following stenting of one upper renal artery branch and percutaneous transluminal angioplasty of the remaining two branches, showing restored flow with no residual stenosis. **(F)** Kaplan Meier Survival Plot for the study cohort.

Donor’s kidney side, warm ischemia time (WIT) (time from intraoperative renal-artery clamping until cold organ flush), CIT (from cold organ flush until the kidney was removed from ice for KT), back-table vascular reconstruction (illustrated as pantaloon/double-barrels Figure 1A, recipient internal iliac artery Y-graft Figure 1B, or end-to-side anastomosis of a small artery to the main artery Figure 1C, vascular anastomosis time, graft-kidney weight, main renal-allograft vessel anastomosis method (either end-to-end anastomosis {EE} to the internal iliac vessel or end-to-side anastomosis {ES} to the external iliac vessel), and anti-thymocyte globulin induction (ATG).

### Immediate Post-Transplant Indicators

DGF (dialysis requirement in the first week post-KT), slow graft function (SGF) (SCr >1.5 mg/dL for more than 10 days following KT), biopsy-proven acute rejection (BPAR), and hospital stay duration.

### Post-KT Discharge Metrics

CMV infection, baseline graft function (mean of last five SCr (mg/dL) following stabilization of allograft function past 1-month post-KT), time to TRAS (days), which was defined as the time from KT till clinical manifestation as per the cTRAS definition, and the number of antihypertensive medications used to control HTN pre- and post-TRAS intervention.

Graft function pre and post-intervention for TRAS (Scr and eGFR calculated using a modified modification of diet in renal disease equation), reduction in number of antihypertensive medications, graft, and patient survival, and graft function at follow-up. Graft failure was labeled when a KTR required maintenance hemodialysis.

### Immunosuppression Protocol

All living KTRs with HLA mismatch >3 and deceased donor KTRs received ATG induction (1 mg/kg body weight for 3 days). For living KTRs with HLA mismatch <3, Simulect induction was administered, while in selective cases with a full HLA match, no induction agent was utilized. All KTRs then received a center-specific triple-drug immunosuppression regimen (tacrolimus 0.2 mg/kg/day in two divided doses, mycophenolate mofetil 1 g BD, and prednisolone 0.4 mg/kg OD), along with concomitant antimicrobial and anti-CMV prophylaxis, with steroids tapered to 5 mg at 3 months.

### CDU Protocol

Study centers KTRs undergo standard CDU before discharge to assess graft vascularity, renal artery peak systolic velocity, and detect any fluid collections. Those with abnormal findings are subjected to sequential monitoring of graft function, incorporating additional imaging tests based on the initial CDU results.

### Surgical Protocols and Techniques: Donor Kidney Selection

The study center’s protocol for living-KT includes laparoscopic procurement of kidneys with a single renal artery (SRA) or the left kidney in cases of bilateral MRA, aiming to maintain a split differential glomerular filtration rate (GFR) discrepancy under 10%. Standard laparoscopic living-donor nephrectomies, KTR-surgeries, and an in-house deceased-organ retrieval were performed according to the established procedures [7, 8].

### Operating Coordination and Anastomosis Strategy

Two senior transplant surgeons (STS) meticulously performed the *ex-vivo* VR-MRA during back-table bench surgery, using 3.5-4X magnification surgical loupes. The VR-MRA was performed over atraumatic silastic catheters using double-armed 7-0 monofilaments in an interrupted fashion.

In cases of live-KT, donor and recipient surgeries were performed in adjacent operating rooms, led by two senior transplant surgeons (STS) with combined experience of over 2000 KT, and assisted by two transplant surgery fellows. Post-retrieval, kidneys were flushed with cold Histidine-Tryptophan-Ketoglutarate solution and subsequently preserved in ice until KT. For living donor-KT, VR-MRA was performed when MRAs were present to ensure optimal perfusion of the graft kidney. However, in a minority of cases where VR-MRA could not be safely performed without kinking the MRA, the additional arteries were implanted separately. In contrast, for deceased donor-KT, MRAs were always implanted with a Carrel’s patch. VR-MRA was required only when the MRAs were too far apart to be included in a single patch or when the MRA were injured during kidney retrieval.

For both live and deceased-KT, the SRA and MRA (after VR-MRA) were preferably anastomosed end-to-end to the Internal Iliac Artery (IIA) when its patency was confirmed. In cases where the IIA was not suitable for anastomosis, such as lumen size discrepancy or atherosclerotic IIA, the SRA or MRA was attached to the External Iliac Artery (EIA) using a punch arteriotomy in an end-to-side (ES) fashion. In deceased-KT, MRAs without VR-MRA were preferably anastomosed on the Carrel patch in ES fashion to the EIA.

### Surgical VR-MRA Techniques

• For two RA with a lumen discrepancy of up to 70:30 and aligned ostial axes, a side-to-side double-barrel/pantaloon technique was employed (Figure 1A).
• In cases where two RA were distantly separated for pantaloon-anastomosis without undue tension, an internal iliac artery (IIA) Y-graft reconstruction was performed using the recipient’s IIA (Figure 1B).
• If the lumen discrepancy falls short of 70:30, the smaller RA was anastomosed in an end-to-side (ES) fashion to the larger RA or anastomosed separately as end-to-end (EE)or ES to the IIA or external iliac artery (EIA), respectively (Figure 1C).
• The senior transplant surgeon (STS) employed customized approaches for complex scenarios involving three or more RA, selecting from or combining the aforementioned techniques (Supplementary Figure S1).
• The renal vein was consistently anastomosed ES to the external iliac vein.

### Management Strategies for cTRAS

All endovascular interventions (EVI) for cTRAS were performed by a single senior interventional cardiologist experienced in about ten thousand percutaneous coronary interventional procedures. Stents were deployed in cases where post-angioplasty residual stenosis exceeded 30%. Drug-eluting stents (DES) were preferred for smaller renal arteries (≤5 mm), while bare metal stents (BMS) were chosen for larger ones (≥5 mm). Success following EVI for cTRAS was defined both clinically and technically. Technical success was indicated by a minimal systolic pressure gradient or clear fluoroscopic evidence of no residual stenosis. Clinically, success was defined as a reduction in SCr by more than 20% or a decrease in atleast one antihypertensive medication within 2 weeks post-intervention. All KTRs diagnosed with cTRAS underwent an initial CDU at 4 weeks post-EVI, followed by surveillance scans every 6 months, and then annually after that. Post-EVI, all KTRs were prescribed dual-antiplatelet therapy (aspirin 75 mg and clopidogrel 75 mg daily) for 1 year, and those experiencing recurrent cTRAS continued the therapy for life.

### Sample Size and Hypothesis

The sample size was determined with the null hypothesis that *ex-vivo* VR-MRA to create a common channel for anastomosis does not increase the risk of cTRAS [9]. With a power of 80%, an alpha level of 5%, and a ratio of four matched controls per cTRAS case, the calculation factored in a 10% probability of exposure in the control group (reflecting the incidence of bilateral MRA in the donor) [10]. A correlation coefficient of 0.2 was chosen to account for a small anticipated effect size and to minimize the risk of Type-II errors, ensuring an adequately powered study. Drawing from a previous study where Y-graft was identified as an independent risk factor for cTRAS (odds ratio = 4.957) [6]. Similar odds were assumed for other VR-MRA techniques involving creating a common channel for anastomosis (e.g., pantaloon technique, end-to-side anastomosis of smaller RA Figure 1A–C). These calculations determined a minimum sample size of 22 cTRAS cases and 88 controls.

### Statistical Analysis

Univariable comparisons of continuous data were conducted using Student's t-test or the Mann–Whitney U test based on data distribution. Categorical data were analyzed using the χ2 test or Fisher’s exact test. The Wilcoxon Signed-Rank and Stuart-Maxwell tests were applied to pre- and post-intervention analyses. Univariable logistic regression was employed for each significant variable to evaluate its association with the outcome, followed by multivariable logistic regression to adjust for potential confounders, thereby generating multivariable odds ratios. The study analysis employed a dual-method analytical framework, integrating multivariable regression analysis with all predictor variables and a bidirectional stepwise selection methodology to validate the significance of VR-MRA as an independent risk factor for cTRAS. The robustness of the logistic regression model was then assessed using the Chi-Square statistic, Pseudo R^2^, Akaike Information Criterion, C-statistic, and Hosmer-Lemeshow test to ensure a reliable statistical assessment of cTRAS predictors. A P-value of <0.050 was considered statistically significant.

## Results

Of the 2,454 KT performed during the study period, 28 KTRs (1.14%) were diagnosed with cTRAS. The average time for the presentation of cTRAS was around 110.07 ± 31.78 days post-KT, with most cases (78.57%) exhibiting stenosis in the juxta-anastomotic region (Figure 1D). This juxta-anastomotic region narrowing was observed in all cases involving VR-MRA and in 45.45% of cases with SRA. (Supplementary Figures S2, S3A, B). Renal allograft dysfunction, marked by elevated SCr, was the primary clinical manifestation in 92.86% of cTRAS cases. Furthermore, over half of cTRAS cases (57.14%) necessitated the usage of ≥2 antihypertensive medications. Clinical features of fluid overload, such as weight gain and pulmonary edema, were present in two KTRs. Beyond the 28 cTRAS cases, three KTRs not included in this study were identified with early-stage TRAS attributed to dissection of EIA.

The etiology of ESRD in cTRAS cases were diabetic nephropathy (28.57%), IgA nephropathy (21.42%), obstructive nephropathy (10.71%), hypertensive nephropathy (7.14%), and autosomal dominant polycystic kidney disease (7.14%). The underlying etiologies remained unidentified in 25% of cTRAS cases.

No significant differences were observed between cTRAS cases and controls in baseline pretransplant and intraoperative parameters, except for the higher occurrence of VR-MRA (53.57%) (p < 0.001) and MRA (60.7%) (p < 0.001) in cTRAS cases. All MRA allografts in the cTRAS cohort underwent VR-MRA, except for two cases from living donor-KT, where the MRAs were implanted separately into the IIA and EIA (Table 1). VR-MRA was performed in 55% of living-KTR and 50% of deceased donor-KTR diagnosed with cTRAS. Postoperatively, slow graft function (SGF) was more prevalent in cTRAS cases (64.28%) compared to controls (36.60%) p = 0.013. Despite a significantly higher rate of SGF in cTRAS cases, both cases and controls recorded a similar baseline line Scr (mg/dL) and eGFR 1-month post-KT. Furthermore, all cTRAS cases exhibited normal CDU results upon discharge following the KT. However, before cTRAS was diagnosed, frequent allograft biopsies were performed in cTRAS cases for prevalent allograft dysfunction. Mild to moderate acute tubular necrosis (ATN) was the most common biopsy finding in 67.85% of cTRAS cases, and 10.71% of cTRAS cases had biopsy-proven acute rejection (Tables 1–3).

**TABLE 1:** Baseline pretransplant, intraoperative, and postoperative characteristics.

KTR characteristics	TRAS-KTR (n = 28)	Non-TRAS-KTR (n = 112)	P-value
**Baseline Pretransplant Parameters**			
Type of Transplant (Live/Deceased KT)	(20/8) (71.4%/28.6%)	(80/32) (71.4%/28.6%)	1.000
Blood Group A	10 (35.7%)	31 (27.7%)	0.842
B	10 (35.7%)	41 (36.6%)	
AB	3 (10.7%)	15 (13.4%)	
O	5 (17.9%)	25 (22.3%)	
Donor age (yrs) (mean ± SD) (median)	44.86 ± 12.10 (43)	41.68 ± 12.53 (43.50)	0.224
Donor BMI (kg/m^2^) (mean ± SD) (median)	23.07 ± 2.97 (22.25)	24.48 ± 4.68 (24.00)	0.127
Donor Sex (Female/Male)	(20/8) (71.4%/28.6%)	(66/46) (58.9%/41.1%)	0.281
KTR age (yrs) (mean ± SD) (median)	37.64 ± 13.71 (37.50)	36.28 ± 11.65 (35.00)	0.630
KTR BMI (kg/m^2^) (mean ± SD) (median)	22.07 ± 3.34 (22.04)	22.61 ± 4.12 (22.50)	0.794
KTR Sex (Female vs. Male)	(5/23) (17.9%/82.1%)	(20/92) (17.8%/82.1%)	1
Pre-KT Haemodialysis Duration (months) (mean ± SD) (median)	25.89 ± 22.77 (12.00)	23.57 ± 26.06 (12.00)	0.661
HLA Mismatch (≤3 vs> 3)	(11/17) (39.3%/60.7%)	(49/63) (43.8%/56.2%)	0.669
Diabetes Mellitus	(8) (28.6%)	(36) (32.1%)	0.716
**Intraoperative Parameters**			
Donor Kidney Side (Left/Right)	(22/6) (78.6%/21.4%)	(99/13) (88.4%/11.6%)	0.216
Warm Ischemia Time (minutes) (mean ± SD) (median)	3.54 ± 2.36 (5.00)	3.69 ± 2.61 (5.00)	0.793
Cold ischemia Time (minutes) (mean ± SD) (median)	180.18 ± 116.50 (125.50)	180.42 ± 196.42 (100.00)	0.047
Anastomosis Time (minutes) (mean ± SD) (median)	30.93 ± 3.68 (30.00)	31.10 ± 2.07 (30.00)	0.623
Living Donor Surgery Operating Time (minutes) (mean ± SD) (median)	194.5 ± 31.37 (190.00)	198.62 ± 28.41 (180.00)	0.768
Donor Kidney weight (grams) (mean ± SD) (median)	141.64 ± 36.98 (138.50)	149.45 ± 34.89 (144.00)	0.302
Multiple Renal Arteries {double RA, triple RA}	(17){12 + 5} (60.7%)	(16){15 + 1} (14.3%)	<0.001
Vascular Reconstruction for Multiple renal arteries (VR-MRA) (a+b + c)	(15) (53.6%)	(9) (8.0%)	<0.001
a. Double Barrel (VR-MRA) (Figure 1A)	9 (32.1%)	5 (4.5%)	<0.001
b. Y-Graft (VR-MRA) (Figure 1B)	4 (14.3%)	2 (1.8%)	0.015
c. End-to-side (VR-MRA) (Figure 1C)	2 (7.14%)	2 (1.78%)	0.18
End-to-end Anastomosis (Graft Implantation to Internal Iliac Artery)	(11) (39.3%)	(67) (60.7%)	0.073
End-to-side Anastomosis (Graft Implantation to External Iliac Artery)	(17) (60.7%)	(45) (40.2%)	0.050
**Postoperative Parameters**			
Antithymocyte Globulin Induction	(18) (64.3%)	(64) (57.11%)	0.493
Slow Graft Function	(18) (64.23%)	(41) (36.6%)	0.008
Delayed Graft Function	(5) (17.9%)	(10) (8.9%)	0.181
Renal Allograft Biopsy	(25) (89.3%)	(45) (40.2%)	<.001
Duration of Post-Transplant Hospital Stay (Days) (mean ± SD) (median)	14.32 ± 6.8 (12)	12.26 ± 6.28 (10)	0.074
Biopsy-proven acute rejection	(3) (10.7%)	(17) (15.2%)	0.764
Baseline SCr (mg/dL) (mean ± SD) (median)	1.30 ± 0.38 (1.30)	1.42 ± 0.49 (1.30)	0.458
Baseline eGFR (mean ± SD) (mL/min/1.73 m^2^) (median)	71.95 ± 27.85 (67.50)	70.80 ± 25.76 (67.50)	0.845
Follow-up SCr (mg/dL) (mean ± SD) (median)	1.35 ± 0.40 (1.35)	1.82 ± 1.49 (1.40)	0.567
Follow-up eGFR (mean ± SD) (mL/min/1.73 m^2^) (median)	70.43 ± 21.29 (69.50)	66.55 ± 32.34 (61.00)	0.446
Patient Survival	(26) (92.9%)	(106) (94.6%)	0.660
Renal allograft Survival	(27) (96.4%)	(102) (91.1%)	0.694

**TABLE 2:** Multivariable regression model.

**Regression with all variables in the model**	**OR (univariate)**	**OR (multivariate)**
Cold Ischemia Time (Minutes)	1.00 (1.00–1.00, p = 0.995)	1.00 (1.00–1.00, p = 0.700)
End-to-side anastomosis	2.30 (1.00–5.50, p = 0.054)	2.08 (0.77–5.80, p = 0.150)
Multiple Renal Arteries	9.27 (3.75–24.11, p< 0.001)	2.00 (0.26–10.47, p = 0.440)
Slow Graft Function	3.12 (1.34–7.63, p = 0.010)	3.55 (1.31–10.49, p = 0.015)
Vascular reconstruction of multiple renal arteries (VR-MRA)	13.21 (4.95–37.69, p < 0.001)	7.43 (1.31–62.43, p = 0.035)
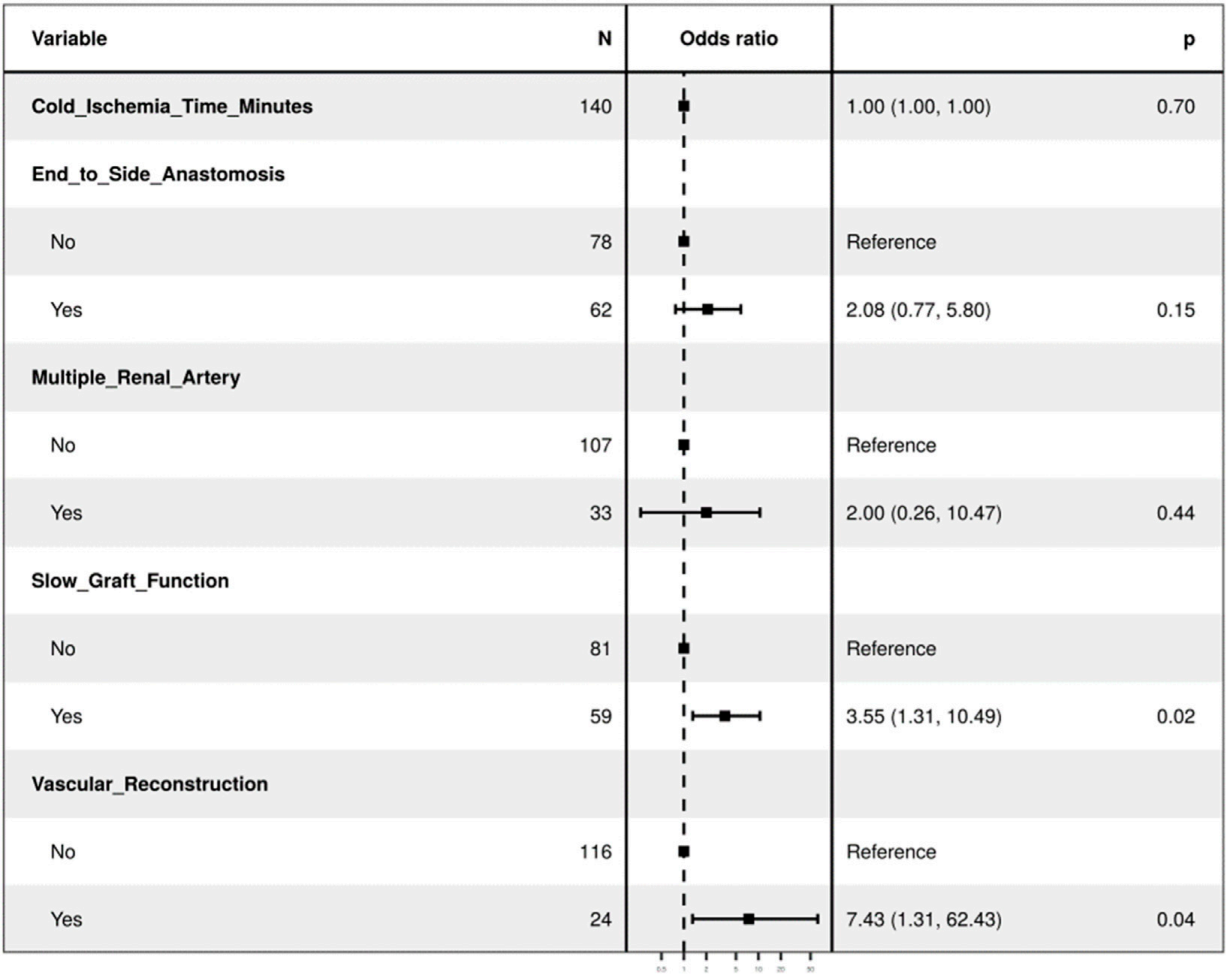		
**Regression with selected variables in the model (Bidirectional Stepwise Selection)**	**OR (univariate)**	**OR (multivariate)**
Cold Ischemia Time (Minutes)	1.00 (1.00–1.00, p = 0.995)	-
End to side- Anastomosis (ES)	2.30 (0.99–5.37, p = 0.054)	2.09 (0.78–5.61, p = 0.145)
Multiple Renal Arteries	9.27 (3.68–23.38, p < 0.001)	-
Slow Graft Function (SGF)	3.12 (1.31–7.39, p = 0.010)	3.66 (1.32–10.12, p = 0.013)
Vascular reconstruction of multiple renal arteries (VR-MRA)	13.21 (4.82–36.18, p < 0.001)	13.51 (4.58–39.88, p < 0.001)
**Regression with selected variables in the model (Bidirectional Stepwise Selection)**	**OR (univariate)**	**OR (multivariate)**
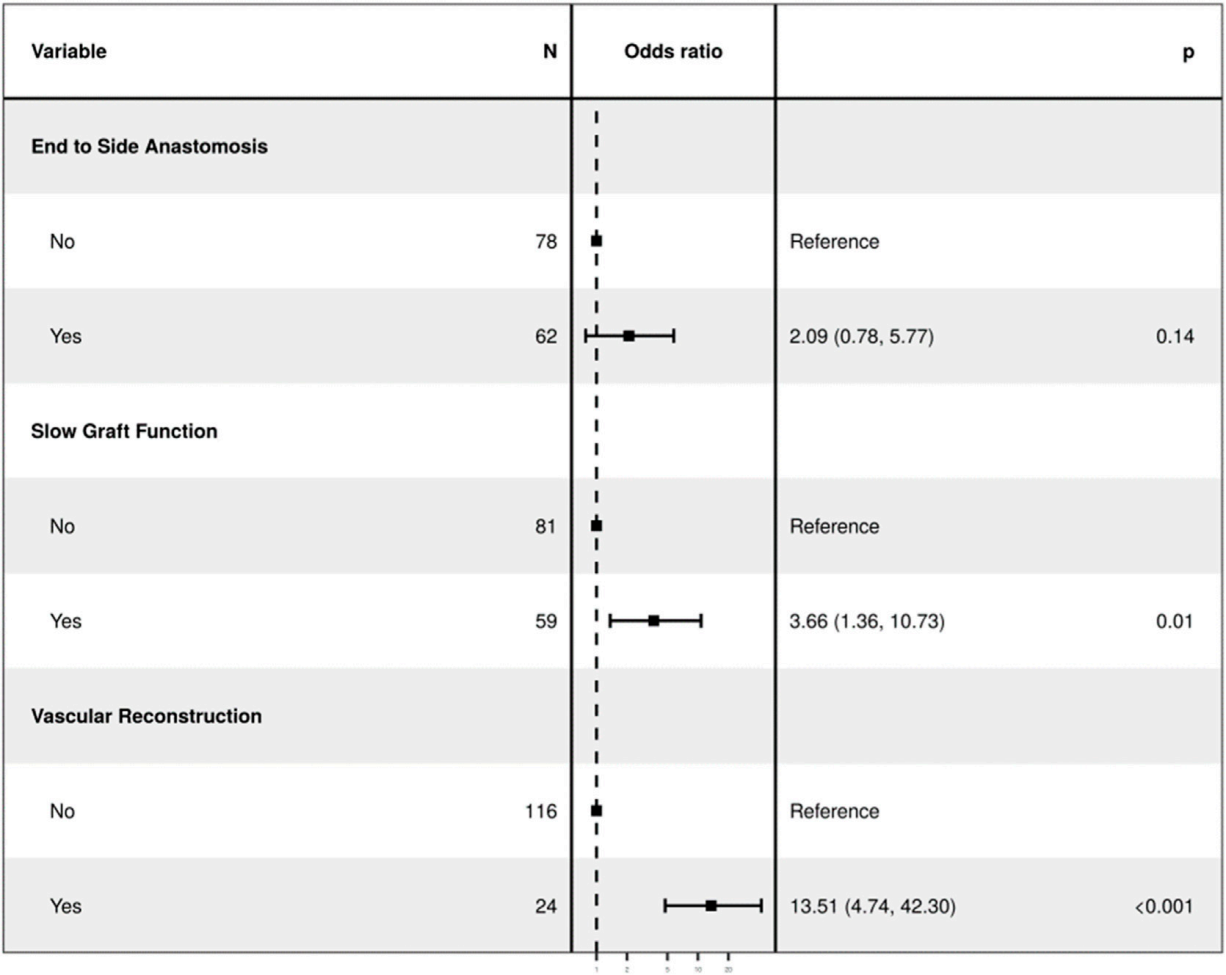		

**TABLE 3:** cTRAS Cases baseline parameters.

TRAS cases parameters	
Time to TRAS (Days) (mean ± SD) (median) (Interquartile Q1-Q3)	110.07 ± 31.78 (101.00) (Q1 90.75-Q3 130 days)
Follow-up Duration in months {Inter quartile range-IQR}	6–180 months (mean-58.89 months median {IQR} = 43{24–67} months)
Number of Antihypertensive medications at TRAS Diagnosis (mean ± SD)	2.46 ± 0.92
Number of Antihypertensive medications at 1 month Post Intervention (mean ± SD)	1.61 ± .057 χ2 = 18.237, p = 0.001
SCr (mg/dL) at TRAS Diagnosis (mean ± SD)	2.06 ± 0.85
SCr (mg/dL) 2-week Post TRAS Intervention (mean ± SD)	1.33 ± 0.36 (p < 0.001)
eGFR at (mL/min/1.73 m^2^) at TRAS Diagnosis (mean ± SD)	48.50 ± 21.15
eGFR at (mL/min/1.73 m^2^) 2-week Post TRAS Intervention (mean ± SD)	69.13 ± 21.87 (p< 0.001)
Biopsy feature	• Mild (n = 17)
• Acute tubular necrosis	• Moderate (n = 5)
• BPAR	• (n = 3) KTRS
VR-MRA- Double Barrel/Y-graft/ES to main RA	9/4/2 = 15
Only Angioplasty	n = 3
Angioplasty + Stenting (BMS)	n = 10
Angioplasty + Stenting (DES)	n = 15
Restenosis in KTR	n = 2

The diagnosis of cTRAS was confirmed through angiography in all cases except for three KTRs (Figure 1E), where magnetic-resonance angiography was employed to diagnose cTRAS following inconclusive CDU findings with a high index of suspicion for cTRAS with graft-dysfunction. N = 27 cases of cTRAS were successfully managed with EVI. EVI predominantly comprised percutaneous transluminal angioplasty (PTA) with stenting in 89.28% of cases. PTA alone was performed in three KTRs. Intravascular imaging using optical-coherence tomography was employed in seven cTRAS cases to optimize EVI. Notably, all EVIs were accomplished without any procedural complications. Recurrent cTRAS, manifesting as in-stent restenosis (ISR), occurred in two KTRs at one and 4 years post-EVI procedures, leading to a reintervention rate of 7.14%. Both these cases were successfully treated with cutting balloon angioplasty and DES. In cTRAS cases, the patency rates following EVI were 92% for PTA with stenting and 100% for PTA alone. Following EVI, significant clinical improvements were observed, including decreased SCr levels and reduced requirement for antihypertensive medications (Table 3). Within the cTRAS-cohort, one fatality was attributed to cTRAS in a KTR who presented with severe graft dysfunction (Scr = 5.2 mg/dL) in a hypertensive crisis, volume-overload, and pulmonary edema, a clinical scenario known as Pickering syndrome. Another fatality in the cTRAS group resulted from COVID-19 infection at 1 year post-EVI. Seven KTRs with cTRAS experienced a diabetes-insipidus-like state following EVI and required conservative therapy, consequently prolonging their hospital stay by 1 week.

Follow-up duration for cTRAS cases varied from 6 to 180 months (mean-58.89 months, median-43 months). Notably, both the cTRAS cases and control groups demonstrated comparable graft and patient survival rates (Kaplan-Meier survival Figure 1F). The study analysis confirms and validates the significance of VR-MRA as an independent risk factor for cTRAS (p < 0.001) in the study cohort (Table 2).

## Discussion

The occurrence of cTRAS significantly increases the risk of allograft loss and mortality among KTRs. However, prompt diagnosis and management of cTRAS can potentially improve patient and graft survival [2, 3, 11]. The risk factors inciting cTRAS in living-KT remain inadequately defined. This study represents the largest single-center experience from Asia, highlighting VR-MRA as a significant independent risk factor for cTRAS in a predominantly living KT program. cTRAS mainly occurred in the juxtanastomotic area, typically around 3.62 ± 1.04 months post-KT, with timely management of cTRAS resulting in graft outcomes similar to those in KTRs without cTRAS.

VR-MRA, as a significant predictor for cTRAS, holds particular importance in the context of evolving transplant practices across the globe and underscores the critical relevance of index study in informing surgical decisions and patient outcomes in Kidney transplantation. With the advancement of laparoscopic kidney retrieval, many transplant surgeons have shown a growing preference for using left kidneys from living donors for KT despite the presence of MRAs, which often necessitates VR-MRA. In the USA, left laparoscopic donor-nephrectomy is the preferred method for KT, with an adoption rate of 86.1%, regardless of the presence of MRA [12]. In contrast, practices in the UK vary; some centers exclusively opt for left laparoscopic donor-nephrectomy, while others prefer kidneys with SRA, as is the current practice at the index center [13]. A few small series have reported a heightened risk of cTRAS in KTRs who received allografts with MRA in living donor-KT [6, 14]. Additionally, studies on TRAS outcomes in both living and deceased donor KT have noted a higher prevalence of allografts with MRA in their TRAS groups. [15, 16]. Furthermore, a small subgroup analysis suggested an elevated risk of cTRAS for both living donor-KT (cTRAS, n = 13) and deceased donor-KT (cTRAS, n = 20) involving MRA that underwent VR-MRA [6, 17]. Meta-analysis amongst KTRs receiving allografts with MRA has also revealed that KTRs receiving allografts with MRAs face significantly higher immediate vascular complications like bleeding and vascular thrombosis, increased DGF, elevated SCr at one/5 years, and decreased 1-year graft survival when compared to KTRs receiving allografts with SRA regardless of donor type (living or deceased-donor) [18, 19]. However, these meta-analyses did not explicitly investigate the occurrence of cTRAS in SRA versus MRA groups and their impact on graft outcomes.

The incidence ratio of cTRAS (1.14%) observed in the index study potentially reflects a falsely low estimate compared to the broader reported range of 1%–23% [3–5, 11]. This discrepancy can be primarily attributed to the absence of routine imaging screening methods for cTRAS at the study center. The existing literature indicates that cTRAS typically manifests within the initial 3–6 months post-KT, with as many as 78% of cTRAS cases exhibiting stenosis primarily in the juxta-anastomotic region of the donor-renal artery [3, 6, 11, 15, 20–25]. The findings of the index study affirm this trend. The juxta-anastomotic region may be prone to altered shear stress-induced endothelial damage due to turbulent renal blood flow (RBF), particularly when the RBF transitions from an SRA to reconstructed MRAs implanted with a single common channel. This juxta-anastomotic region might also be affected by stretching or redundancy in the reconstructed arteries after the final placement of the renal allograft in the KTR, potentially leading to localized endothelial injury and the development of neointimal-hyperplasia (IH) in the juxta-anastomotic region [26, 27]. Immunological factors like allograft rejection and Class-II de-novo donor-specific antibodies (cutoff mean fluorescence intensity of over 300) have been proposed as potential risk factors for TRAS [11, 22, 28]. The predominant localization of cTRAS to the juxta-anastomotic region, as observed in numerous studies, including ours, strongly suggests that the primary etiological factor is altered hemodynamics rather than an immunological response [29, 30]. Typically, immunological factors would be expected to cause more widespread endothelial damage than a focal endothelial injury. Although the study center did not routinely screen for de-novo DSA in all KTRs, the rates of allograft rejection and HLA mismatches were non-significant (Table 1).

Poiseuille’s law underscores the exponential influence of vascular-radius on the RBF rate (Q = ΔPπr⁴/8ηl), where even modest luminal reductions due to IH (5%–15%) can significantly decrease RBF by 18.5%–47.8% (Supplementary Figures S2A, B, S4, S5). The impact of IH causing luminal reduction is more pronounced in allograft implanted with VR-MRA, particularly when the same thickness of IH extends from larger SRA to smaller MRAs in the juxtanastomotic region, leading to a substantial reduction in luminal diameter, thereby significantly reducing RBF. Elevated blood pressure is necessary to maintain RBF in such circumstances of reduced luminal diameter, ultimately leading to a vicious cycle of increased turbulence and low shear stress on endothelial cells in the juxta-anastomotic region, exacerbating endothelial damage by promoting the release of prothrombotic factors (Supplementary Figures S2A) [2, 26, 27, 30]. A recent randomized clinical trial reinforces this mechanistic understanding by demonstrating that low-dose aspirin (100 mg) effectively reduces cTRAS development amongst KTRs [31]. Aspirin prevents microthrombi formation by inhibiting platelet aggregation in areas of abnormal shear stress, underscoring the critical role of platelets in the pathogenesis of cTRAS [32].

The high procedural success rate of EVI at the study center reinforces its established efficacy as the preferred therapeutic method for treating cTRAS [33, 34]. KTRs who underwent EVI at the index study center demonstrated significant improvements in SCr and reduced reliance on antihypertensive medications, paralleling the long-term graft and patient survival observed in KTRs without cTRAS (Table 3). The efficacy of EVI in managing cTRAS largely stems from the early detection of cTRAS and the expertise of the interventional team. Moreover, the study center’s adoption of optical coherence tomography for guiding EVI has refined the therapeutic approach, contributing to advancements in this domain [35].

The index study has certain limitations. Firstly, the study’s design does not allow for definitive causality establishment, a limitation of case-control studies. The limited cohort size presented a constraint in conducting extensive subgroup analyses between living donor-KT and deceased donor-KT. The study’s emphasis on VR-MRA within a small sample size may have reduced its power to evaluate other risk factors for cTRAS. While VR-MRA emerged as an independent risk factor for cTRAS in our study, we also recognize that a smaller luminal diameter at the graft implantation site, irrespective of VR-MRA, may contribute to the risk of cTRAS. However, we could not perform a subgroup analysis due to the limited number of cTRAS cases involving MRAs implanted separately without VR-MRA (n = 2). To definitively determine whether the primary factor driving turbulence and the subsequent occurrence of cTRAS is the luminal diameter or the presence of VR-MRA, a larger study that includes measurements of the minimum diameter at the arterial anastomosis across MRAs undergoing VR-MRA versus those implanted separately would be essential. Such a study would clarify the specific contributions of smaller luminal diameter and VR-MRA to the risk of cTRAS. Additionally, using retrospective odds ratios for sample size calculation may have limited the precision in capturing a full spectrum of effect sizes. Moreover, the predominance of data from living donor-KT in the index study could limit the applicability of the findings to deceased donor-KT, which often involves MRA allografts implanted on a Carrel patch without VR-MRA. Lastly, variability in CDU techniques due to operator differences could have led to inconsistent cTRAS detection, especially in less obvious clinical cases. Considering all these factors, the study’s findings should be interpreted with caution. The study’s strength is evidenced by enhanced validity achieved through a meticulous study design that includes precise power estimation. By meticulously matching cases to controls, the study controlled for confounding factors, reducing selection bias and biases due to surgical variations. Thereby enhancing the representativeness and applicability of the findings, particularly in the context of living donor-KT.

The predominance of cTRAS, particularly in the juxta anastomotic region within the first 6 months after KT, underscores the need for early intervention. We recommend routine CDU screenings during this critical period, especially for KTRs with VR-MRA, to enhance graft and patient survival, enabling early identification and treatment of cTRAS.

## Data Availability

The raw data supporting the conclusions of this article will be made available by the authors, without undue reservation.

## Abbreviations

ATN, Acute tubular necrosis; BMS, Bare metal stents; CDU, Color Doppler ultrasound; CIT, Cold ischemia time; cTRAS, Clinical transplant renal artery stenosis; DES, Drug-eluting stents; DGF- Delayed graft function; DM, Diabetes mellitus; ECD, Expanded criteria donors; EIA, External iliac artery; ES, end-to-side anastomosis; ESRD, End-stage renal disease; EVI, Endovascular interventions; eGFR, Estimated glomerular filtration rate; IH, Neointimal Hyperplasia; IIA, Internal iliac artery; ISR, In-stent restenosis; KT, Kidney transplantation; KTRs, Kidney transplant recipients; MRA, Multiple renal arteries; PTA, Percutaneous transluminal angioplasty; RA, Renal Artery; RBF, Renal blood flow; Scr, Serum creatinine; SGF, Slow graft function; SRA, Single renal artery; STS, Senior transplant surgeon; TRAS, Transplant renal artery stenosis; VR-MRA, *Ex-vivo* back-table vascular reconstruction of multiple renal arteries.
