# Supplementary material for: Vascular Reconstruction of Multiple Renal Arteries—A Risk Factor for Transplant Renal Artery Stenosis: Insight From a Matched Case-Control Study
Source: Transpl Int. 2024 Nov 7;37:13298. doi: 10.3389/ti.2024.13298 (PMC11580035; doi:10.3389/ti.2024.13298)
Supplement: Supplementary file 1 [file DataSheet1.docx]

**Figure S1**- Triple Vessel anastomosis


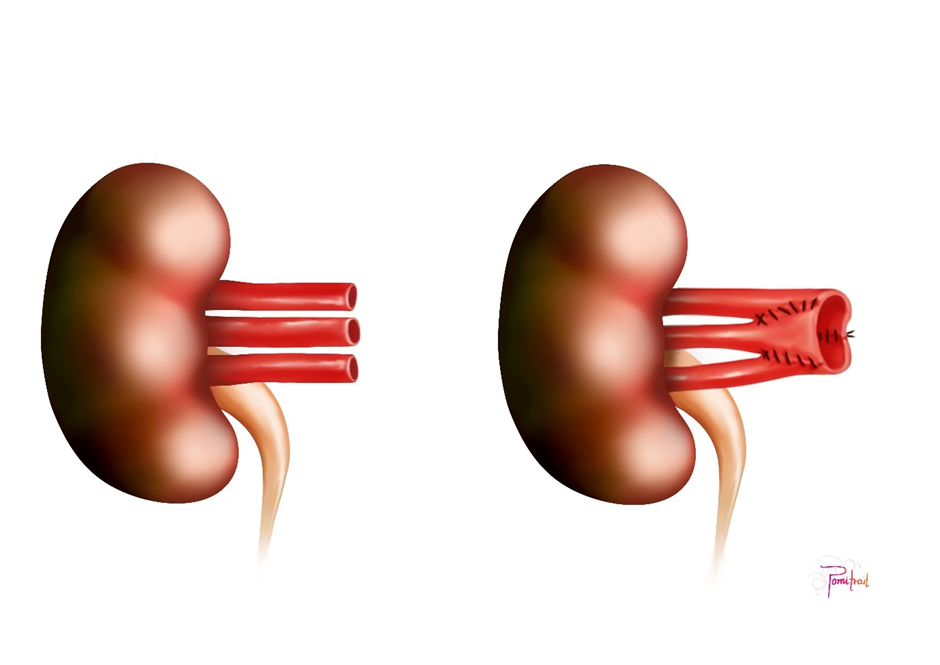


**Figure S2**- Juxta-anastomosis Region with Pantaloon Technique


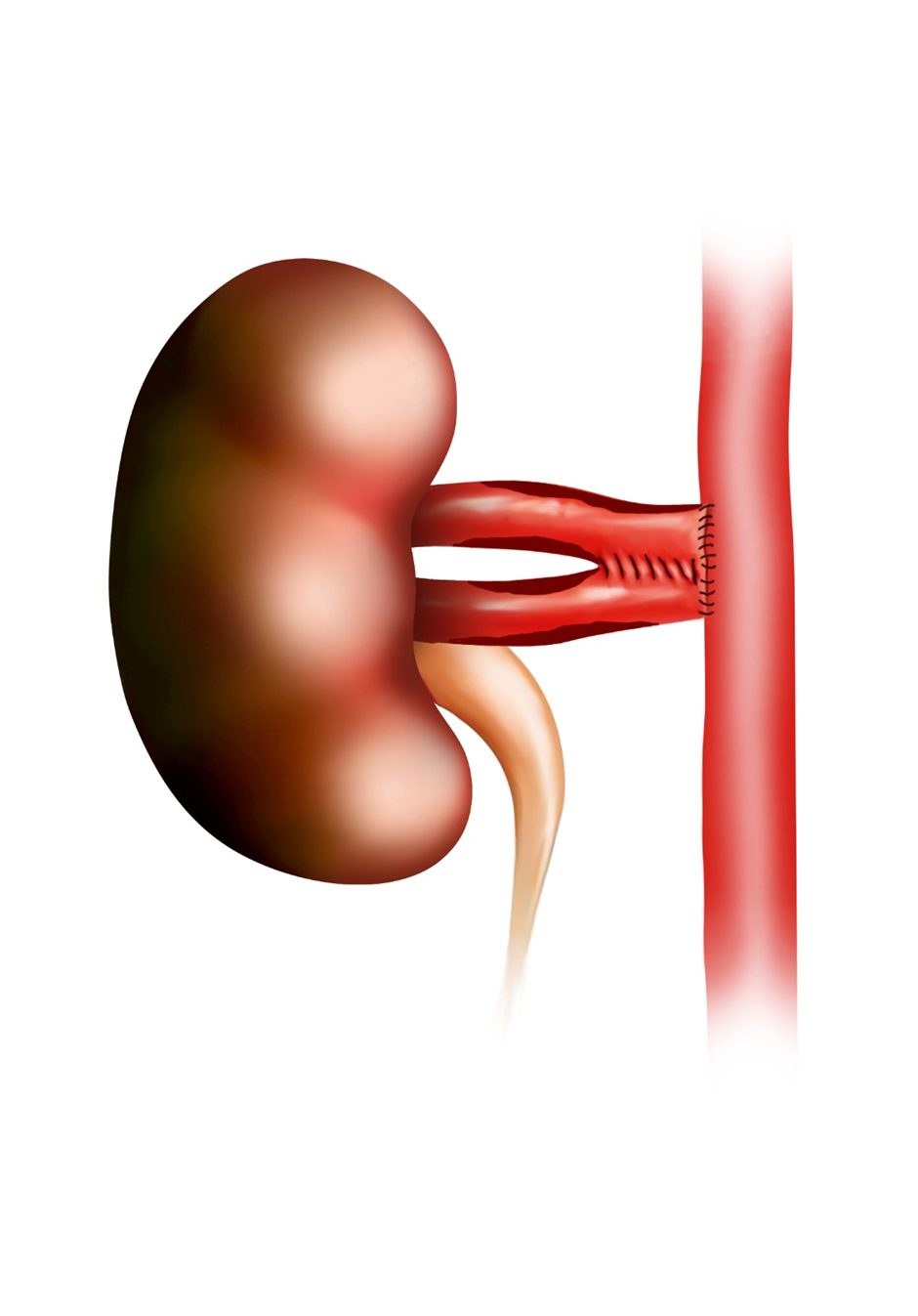


**FigureS3a**- Juxta anastomotic region with Y grafts


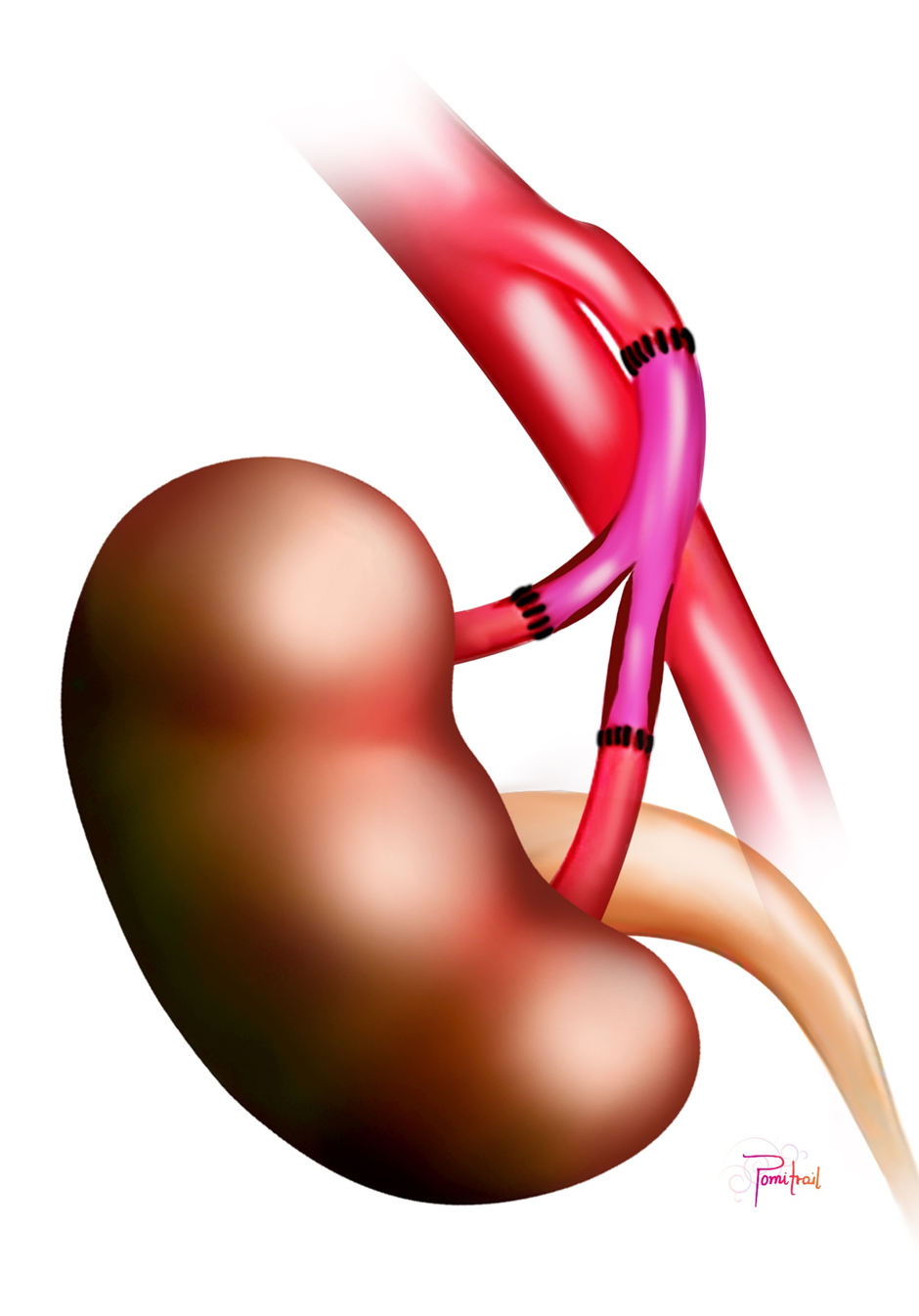


**FigureS3b**- Juxta anastomotic region with Y grafts


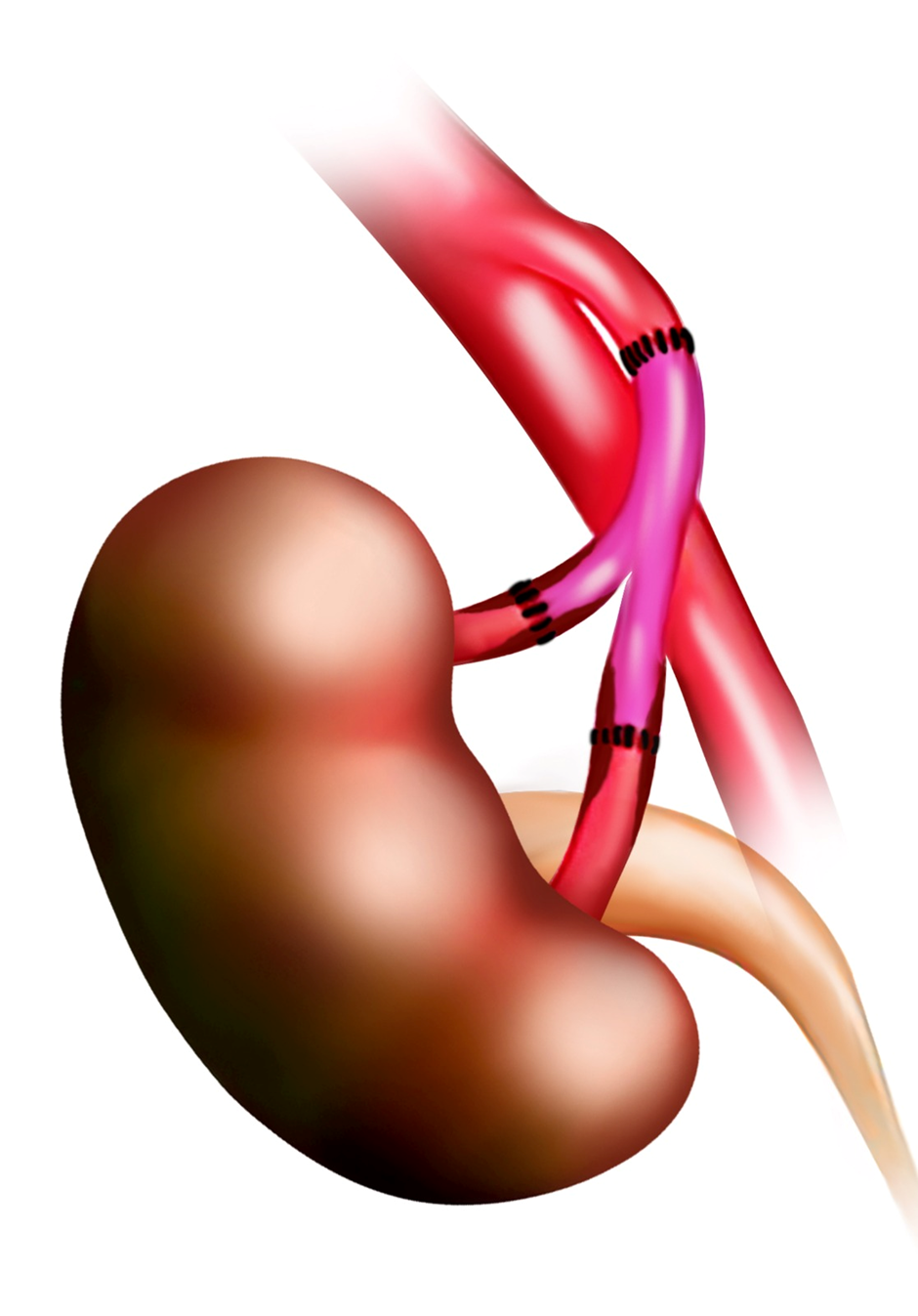


**Figure S4-** Blood Flow rate and % reduction in renal artery radius - Poiseuille's Law


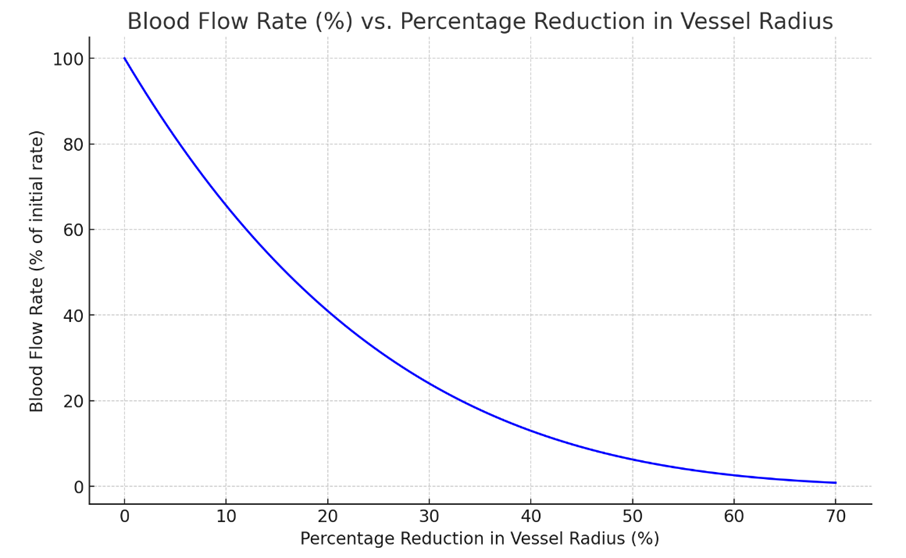


**Figure S5**- Blood Flow rate and Intimal hyperplasia causing a reduction in main and smaller arteries radius - Poiseuille's Law (First smaller artery- 70% of the main artery's radius, second smaller artery-60% of the main artery's radius)


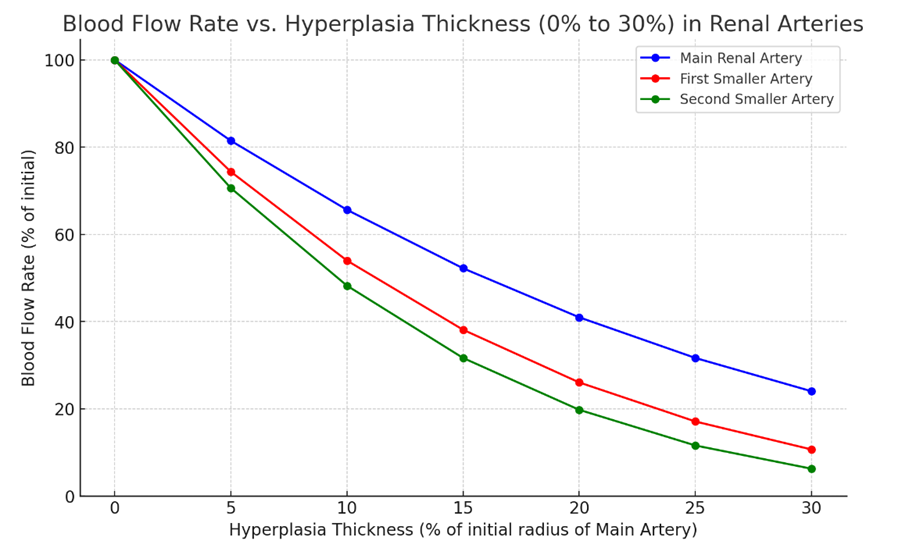


**Capsule Sentence Summary**

This case-control study identified ex-vivo arterial vascular reconstruction of multiple renal arteries as a significant risk factor for clinical transplant renal artery stenosis in kidney transplantation. Timely percutaneous endovascular intervention resulted in favorable long-term graft and patient survival.
